# Dataset to delineate changes in association between Akt1 and its interacting partners as a function of active state of Akt1 protein

**DOI:** 10.1016/j.dib.2017.05.040

**Published:** 2017-05-25

**Authors:** Nutan Gupta, Shweta Duggal, Noor Jailkhani, Samrat Chatterjee, Kanury V.S. Rao, Ajay Kumar

**Affiliations:** a International Centre for Genetic Engineering and Biotechnology (ICGEB), Aruna Asif Ali Marg, New Delhi 110067, India; b Drug Discovery Research Center (DDRC), Translational Health Science and Technology Institute (THSTI), NCR Biotech Science Cluster, 3rd Milestone, Faridabad-Gurgaon Expressway, Faridabad 121001, Haryana, India

**Keywords:** Akt1, SILAC, Interactome, Affinity purification, Mass spectrometry

## Abstract

Akt1 is a multi-functional protein, implicated in multiple human solid tumors. Pertaining to its key role in cell survival, Akt1 is under focus for development of targeted therapies. Functional diversity of Akt1 is a result of its interactions with other proteins; which changes with changing context. This investigation was designed to capture the dynamics of Akt1 Interactome as a function of its active state. Delineating dynamic changes in association of Akt1 with its interactors could help us comprehend how it changes as a function of inhibition of its active form. Similar information on changes in Akt1 interactome as of now is not well explored. Akt1 expressing HEK293 cells were cultured in light and heavy labeled SILAC media. Normal lysine and arginine were incorporated as light labels while for heavy labeling the isotopes were 8 and 10 Da heavier. Light labeled cells represented the indigenous state of Akt1 interactome while heavy labeled cells represented Akt1 interactome in presence of its allosteric inhibitor, MK-2206. Equal number of cells from both conditions were pooled, lysed and subjected to Affinity Purification coupled to Mass Spectroscopy (AP-MS). Additionally, SILAC labeling aided in quantitative estimation of changing association of a number of proteins which were common to the two experimental conditions, with Akt1. Data are available via ProteomeXchange with identifier PXD005976.

**Specifications Table**

**Table:** 

Subject area	*Biology*
More specific subject area	*Proteomics*
Type of data	*Mass Spectrometry Raw Files, figure*
How data was acquired	*Mass Spectroscopy, AB Sciex 5600 Triple TOF*
Data format	*Raw and analyzed*
Experimental factors	*Stable Akt1 expressing HEK293 cells were treated with MK-2206 to delineate changes in Akt1 Interactome as a function of its active state*
Experimental features	*SILAC labeling, LysC – Tryptic Digestion, peptide separation through nano-LC and MS/MS analysis on AB SCIEX 5600 triple-TOF mass spectrometer*
Data source location	*New Delhi, India*
Data accessibility	*Data is within this article and available in a Public repository via ProteomeXchange with identifier ProteomeXchange:*PXD005976

**Value of the data**

• We delineate the indigenous interacting partners of HA-tagged Akt1 protein in HEK293 cells.
• Akt1 protein interactors were extracted from HEK293 cells both in presence and absence of its allosteric inhibitor (MK-2206).
• Our dataset compliments the existing list of Akt1 interactors by validating the already reported interactors and reporting new interacting partners.
• Further experiments on Akt1 interactors, which are dependent on its kinase activity, can add to the existing knowledgebase on Akt1 signaling.

## 1 Data

**Fig. 1:**
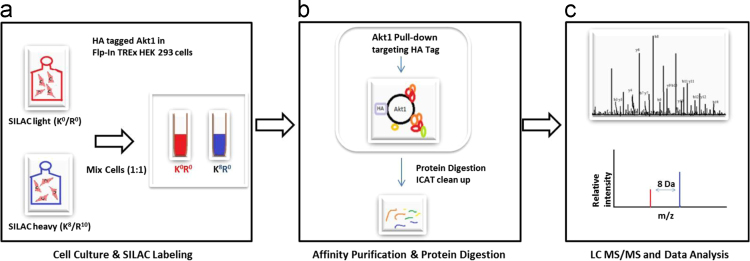
Schematic overview showing the experimental workflow.

HA-tagged Akt1 expressing HEK293 cells were SILAC labeled to capture changes in association of Akt1 interacting partners with Akt1 as a function of its active state. Four biological replicates were processed and analyzed. Affinity purified samples were double digested followed by acquisition and the results were obtained as 4 RAW file pairs (wiff and corresponding wiff.scan files) from AB SCIEX 5600 triple TOF instrument. Wiff files were submitted to protein pilot software version 4.0 and resulted in 16 protein pilot group files (4 group files per biological replicate). The experimental overview is as shown in Fig. 1. Data is publicly available via ProteomeXchange with identifier ProteomeXchange: PXD005976.

## 2 Experimental design, materials and methods

### 2.1 Generation of stable cell line

Using gateway cloning technology (Invitrogen), Akt1 ORF (GE Dharmacon) was shuffled into modified destination vector (pcDNA/FRT/TO; a kind gift from Dr. Matthias Gstaiger, Institute of Molecular Systems Biology, ETH Zurich, Zurich, Switzerland) by homologous recombination. The expression vector was co-transfected with pOG44 recombinase in Flp-In T-REx HEK293 cells, stably expressing the tetracycline (Tet)-repressor. Transfection was facilitated in the presence of Xtremegene 9 transfection reagent (Roche), as per supplier׳s instructions. Cells, stably expressing the construct, were selected in presence of 75 µg/ml hygromycin B for 15–20 days; and later pooled to expand.

### 2.2 Labeling and drug treatment

SILAC (stable isotope labeling in cell culture) medium was supplemented with either normal (light) isotopes of both lysine (Lys^0^) and arginine (Arg^0^) [40 µg/ml of normal L-lysine and 200 µg/ml of normal L-arginine] or heavy isotopes (Lys^8^ and Arg^10^) of the same amino acids [52.22 µg/ml of (^13^C_6_^15^N_2_) L-lysine and 63.42 µg/ml of (^13^C_6_^15^N_4_) L-arginine] [1]. Dialyzed FBS was used during labeling to ensure that the added labeled amino acids were the exclusive source. Akt1 expressing HEK293 cells were cultured for at least 5 cell doublings at 37 °C, 5% CO_2_ to allow full incorporation of the labeled amino acids. Light and heavy labeled Akt1 expressing cells were sub-cultured separately for relative quantitation of protein expression, in the presence and absence of Akt1 inhibitor (MK-2206) [2]. At approximately 70% confluency, Tet was added to the medium, to a final concentration of 1 µg/ml. MK-2206 (5 µM) was supplemented to heavy labeled Akt1 expressing cells after 24 h and cells were continued in culture for another 16–20 h. Finally both heavy and light labeled cells were trypsinized and counted by trypan blue staining and saved as pellet at −80 °C, until used.

### 2.3 Immunoprecipitation of Akt1 and interacting proteins

Light and heavy labeled Akt1 expressing cells were pooled in 1:1 ratio (in equal numbers) and lysed by re-suspending in lysis buffer (150 mM NaCl; 50 mM Tris–HCl pH 7.5; 1% NP-40; 1x protease inhibitor cocktail and 0.1 M PMSF) [3]. Cleared cell lysate was mixed with 100 µl of anti-HA agarose beads and kept for incubation at 4 °C. HA-tagged Akt1 was eluted with HA peptide (250 µg/ml), as per manufacturer׳s instructions (Thermo Scientific). Akt1 interactors were pulled-down in a total of 3 elution steps, pooled and lyophilized, until further use.

### 2.4 Protein digestion and desalting for mass spectrometry

Elution sample was digested with LysC and Trypsin proteases in tandem to ensure complete digestion and minimize mis-cleavages. Lyophilized samples were dissolved in 20 µl dissolution buffer (0.5 M triethyl ammonium bicarbonate; Sciex, Digestion Kit) and 1 µl denaturing reagent (2% SDS; Sciex, Digestion Kit). Cysteine bonds were reduced for 1 h at 60 °C with 2 µl reducing agent (5 mM Tris-2-carboxyethyl phosphine; Sciex, Digestion Kit). The samples were kept for incubation with 1 µl cysteine blocking agent (10 mM methyl methane thiosulfonate; Sciex, Digestion Kit) at RT in dark for 30 min. Five microlitres of 0.1 µg/µl LysC was added and the samples were incubated at 37 °C for 4 h, with enzyme to substrate ratio of 1:40 (w/w). The samples were briefly centrifuged and supplemented with 5 µl of 0.1 µg/µl trypsin; enzyme to substrate ratio of 1:20 (w/w). Incubation was continued at 37 °C for another 12–16 h. Digestion was quenched by adding a drop of formic acid (FA) to the sample tube. The digested samples were lyophilized, re-dissolved in 0.1% FA and subjected to pre-equilibrated C-18 spin columns (Waters) under gravitational force. After 10 washes with 100 µl 0.1% FA, peptides were eluted – once with 100 µl of 40% acetonitrile (ACN) in 0.1% FA and twice each with 100 µl of 60% ACN in 0.1% FA. The three eluates were pooled, and lyophilized.

### 2.5 Cation exchange chromatography

To remove any left-over salts or detergents, peptide samples were subjected to cation exchange chromatography. The cartridge assembly was fixed and prepared; by washing it once with methanol. Ammonium formate (500 mM) was used to condition the cartridge followed by equilibration with 2 washes of 5 mM ammonium formate. The lyophilized sample was re-suspended into 500 µl of 5 mM ammonium formate (pH 2.5) and gently vortexed to mix. The sample was loaded drop-wise onto the cartridge and allowed to flow through, under gravitational force. Following 3 washes with 5 mM ammonium formate, the sample was eluted twice with 400 µl of 500 mM ammonium formate per elution step and lyophilized.

### 2.6 Protein identification by mass spectrometry

Peptides were analyzed by nano flow liquid chromatography on a nanoLC-nanoflex system (Eksigent Technologies, AB SCIEX) coupled to a triple TOF 5600 Mass Spectrometer (AB SCIEX; Concord, Canada). TripleTOF™ 5600 System employs both quadrupole and time-of-flight for mass analysis.

Each fraction was dissolved in 12 μl of 1A buffer (98% water, 2% ACN and 0.1% FA). Injections of 10 µl each were picked up by auto sampler׳s 10 µl loop and directly loaded on a nano-LC trap column. The peptides were trapped and run through loading channel for 40 min at flow rate of 15 µl/min and then eluted out from the analytical column (chromolith column-particle size 5 µm, length 15 cm, 75 µm ID) with constant flow rate of 550 nl/min. The analytical column was directly mounted on electro spray ion source. Peptides were injected into mass spectrometer by using 10 μm SilicaTip and the eluted peptides were monitored with following ion source parameters- IHT-150 degrees, a spray voltage set at 2.2 kv, GS1=20, curtain gas=25. The linear gradient of 5% solvent B (98% ACN, 2% water and 0.1% FA) to 50% solvent B was run for 67 min, followed by linear 50–90% for 8 min. Prior to next run, column was regenerated by washing with 90% solvent B for 10 min and then equilibrated with 5% solvent B for 15 min.

MS data was acquired in information-dependent acquisition mode using Analyst QS 1.5 software (ABSciex). Mass-spectra were recorded in ‘positive-ion’ and “high-sensitivity” mode. LC-MS/MS analysis was performed using TOF-MS survey scan from 350 m/z to 1250 m/z in a scan time of 500 ms, followed by fragmentation of 15 most abundant ion peaks. Accumulation time was set to 100 ms. Rolling collision energy was automatically controlled by information dependent acquisition rolling collision energy parameter script. Selection parameter included for the parent ion to be fragmented was intensity, where ions had to be greater than 120 cps, mass tolerance of 50mDa, with a charge state of +2 to +5. The ions, once fragmented, were excluded from further fragmentation for 12 seconds.

### 2.7 Data processing in protein pilot

Automatic data analysis (MS and MS/MS) and database searching with wiff files obtained from LC-MS/MS analysis with HA enriched samples were conducted against uniprot_human swissprot database (Release April 2016) using Protein Pilot software (version: 4.0, revision no. 148085) with the Paragon method. Following search parameters were selected: Homo sapiens as species, LysC, Trypsin, as enzyme categories for different runs, two missed cleavages allowed; with cys alkylation as methyl methanethiosulphonate. Identification and SILAC (Lys^8^, Arg^10^) as sample types; and the “Search Effort” parameter “Thorough ID”, which gives us a broad search of various protein modifications. Identification and quantification of differentially expressed proteins was strictly checked employing following filters – (1) Auto Bias correction for heavy to light ratio. (2) Threshold of 1% accepted global false discovery rate from fit (G-FDR-fit) proteins and peptide spectrum matches (PSMs); (3) Minimum protein confidence threshold cut-off of 95%; (4) A protein was included in analysis only if atleast a single peptide for the same was identified in all four biological replicates.

The mass spectrometry proteomics data have been deposited to the ProteomeXchange Consortium via the PRIDE [4] partner repository with the dataset identifier PXD005976.
